# Hearing loss in the royal Norwegian navy: a cross-sectional study

**DOI:** 10.1007/s00420-014-0988-8

**Published:** 2014-10-07

**Authors:** Kaja Irgens-Hansen, Erlend Sunde, Magne Bråtveit, Valborg Baste, Gunnhild Oftedal, Vilhelm Koefoed, Ola Lind, Bente Elisabeth Moen

**Affiliations:** 1 Department of Global Public Health and Primary Care, Research Group for Occupational and Environmental Medicine, University of Bergen, Årstadveien 21, 5009 Bergen, Norway; 2 Faculty of Technology, Sør-Trøndelag University College, EC Dahls Gate 2, 7030 Trondheim, Norway; 3 Royal Norwegian Navy Medical Services, Haakonsvern, 5886 Bergen, Norway; 4 Department of Audiology, Haukeland University Hospital, Jonas Liesvei 65, 5021 Bergen, Norway

**Keywords:** Audiometry, Hearing conservation, Hearing loss, Noise exposure, Noise-induced hearing loss

## Abstract

**Objectives:**

Prior studies have indicated a high prevalence of noise-induced hearing loss (NIHL) among Navy personnel; however, it is not clear whether this is caused by work on board. The present study aimed to assess the prevalence of hearing loss among Navy personnel in the Royal Norwegian Navy (RNoN), and to investigate whether there is an association between work on board RNoN vessels and occurrence of hearing loss.

**Methods:**

Navy personnel currently working on board RNoN vessels were recruited to complete a questionnaire on noise exposure and health followed by pure tone audiometry. Hearing loss was defined as hearing threshold levels ≥25 dB in either ear at the frequencies 3,000, 4,000 or 6,000 Hz. Hearing thresholds were adjusted for age and gender using ISO 7029.

**Results:**

The prevalence of hearing loss among Navy personnel was 31.4 %. The work exposure variables: years of work in the Navy, years on vessel(s) in the Navy and years of sailing in the Navy were associated with reduced hearing after adjusting for age, gender and otitis as an adult. Among the work exposure variables, years of sailing in the Navy was the strongest predictor of reduced hearing, and significantly reduced hearing was found at the frequencies 1,000, 3,000 and 4,000 Hz.

**Conclusions:**

Our results indicate that time spent on board vessels in the RNoN is a predictor of reduced hearing.

## Introduction

Noise-induced hearing loss (NIHL) is considered to be one of the most prevalent work-related diseases worldwide. A worldwide analysis states that 16 % of disabling hearing loss in adults is attributable to occupational noise exposure (Nelson et al. 2005).

Studies on occurrence of hearing loss among Navy personnel are infrequent, and to our knowledge, no studies have been published recently (Trost and Shaw 2007; Wolgemuth et al. 1995). Previous studies from the USA have reported noise exposure to be the most prevalent occupational health hazard in the US Navy (Bohnker et al. 2002b), and deteriorated hearing thresholds have been found among 29 % of Navy personnel (Wolgemuth et al. 1995).

Prior studies on hearing loss among Navy personnel have primarily been based on data collected with the purpose of describing and monitoring effects of hearing conservation programs, and hearing has not always been examined systematically in these studies. Few studies have included strict protocol-based measurements, and they have not always considered other potential causes of hearing loss, as for instance non-occupational noise exposure, prior ear disease or exposure to ototoxic medication. The relationship between hearing loss and work on the vessels has not been clearly documented in prior studies.

Navy operations at sea cause noise levels on board RNoN vessels that are higher than recommended limit values (Irgens-Hansen et al. 2013; Koefoed 2011), and in a RNoN study on health and work environment, self-reported prevalence of reduced hearing was 24 % (Moen et al. 2008).

The aim of this study was to assess the prevalence of hearing loss and examine the association between work on board vessels in the RNoN and hearing loss among Navy personnel.

## Methods

### Study population

From April 2012 to June 2013, Navy personnel currently working on board RNoN vessels were asked to participate in a cross-sectional study on noise and hearing by completing an audiometric test and a questionnaire on noise exposure and health. The Navy personnel recruited included officers, enlisted personnel and civilians; 99 % were Caucasians. The total number of Navy personnel fluctuates, and a complete list was not possible to obtain; however, 938 (of the approximately 948 Navy personnel counted at the beginning of the project period) were asked to take part. Information about the study was given in plenary by the management on board each vessel and was also provided through a written letter handed out prior to examination. The study was carried out by trained personnel at the two naval bases (Bergen and Sortland), supervised by a researcher from the University of Bergen. A total of 581 participants were examined in Bergen, while 191 were examined in Sortland.

### Questionnaire

The questionnaire comprised questions regarding occupational and non-occupational factors which could possibly induce hearing loss (Table 1). This included questions about work history, current and prior noise exposure at work and during leisure time, use of hearing protection, general and ear-related medical history, use of ototoxic medication, diving, exposure to ototoxic chemicals, smoking and use of moist snuff. The completed questionnaires were assessed by the personnel who examined the hearing, and participants were asked to clarify ambiguous or missing answers.

**Table 1:** Questions and response alternatives in a questionnaire about noise exposure and health given to Navy personnel in Norway, 2012–2013

	Question	Response alternatives	
Work history	Working position	Free text^b^	
	Years of work in the Navy	Number of years^a^	
	Years on vessel(s) in the Navy	Number of years^a^	
	Years of sailing in the Navy	Number of years^a^	
Current and prior occupational noise exposure	Have you been exposed to impulse noise (explosions etc.) in your work in the Navy without using hearing protection?	Yes/no	Number of times^a^
	Have you had temporary reduced hearing, fullness or ringing in the ears after being noise-exposed during the last year?	Yes/no	Number of times^a^
	Have you used/do you use hearing protection in high noise areas on board vessels in the Navy in these periods?	2010–2012 2000–2009 <2000	Yes, most of the time Sometimes No Of no relevance
	Do you use hearing protection while shooting?	Yes, most of the time Sometimes No	
Current and prior non-occupational noise exposure	Have you been hunting/are you hunting?	Yes/no	Number of seasons^a^
	Do you use hearing protection while hunting?	Yes, most of the time Sometimes No	
	Number of gunshots last year (in the Navy, hunting and sports)	Number of shots^a^	
	Have you played/do you play in a band?	Yes/no	Number of years^a^
	How often do you attend concerts/disco etc. playing loud music?	Weekly^c^ Sometimes/month^c^ Sometimes/year Seldom/never	
	Do you currently use Mp3 player etc. with plugs/phones?	>6 h/week^d^ 3–6 h/week^d^ 1–2 h/week Seldom/never	
Medical history	Have you ever had any of these diseases?	Heart disease Hypertension Diabetes, type 2	Yes No
	Did you have otitis as a child (0–17 years)?	Yes/no/I don’t know	
	Have you had otitis as an adult (from the age of 18 years)?	Yes/no/I don’t know	
	Have you ever been hospitalized due to head injury?	Yes/no	
	Have/had any in you closest family reduced hearing?	Mother/father/children/siblings/none close	
	Have you used ototoxic medication earlier (diuretics, broad spectrum antibiotics, cytotoxins)?	Yes/no/I don’t know	
Other occupational or non-occupational exposure	Have you been diving?	Yes, professional in the Navy Yes, professional outside the Navy Yes, leisure diving No, never	
	Have you had ear damage following diving (being treated in pressurized tank due to the ear damage)?	Yes/no	
	How often do you work with organic solvents (paint/washing with thinner)?	Daily Weekly Monthly Seldom/never	
	Have you smoked/do you smoke?	Yes, daily Sometimes Earlier No	
	Have you used moist snuff/do you use moist snuff?	Yes, daily Sometimes Earlier No	

^a^Continuous variables were grouped by quartiles

^b^The alternative “working position” was grouped into seven job categories

^c^The alternatives “weekly” and “sometimes/month” were merged due to low numbers to the alternative “≥sometimes/month”

^d^The alternatives “>6 h/week” and “3–6 h/week” were merged due to low numbers to the alternative “≥3 h/week”

### Pure tone audiometry

A stepwise test protocol was developed in cooperation with an otolaryngologist and was followed by the personnel performing the audiometry. A checklist was used to ensure that all steps in the procedure were followed. Otological examination was performed prior to audiometry. In cases of complete ear canal obstructions, cerumen was removed and a new appointment was made at least one week later. Pure tone audiometry was done using Interacoustics AD226 with Amplivox Audiocups or Peltor earphones with a lower test limit of −10 dB, or with Welch Allyn GSI with TDH 39 P earphones with a lower test limit of +10 dB. Background noise in the two booths used was measured (15 s) with Brüel & Kjaer sound level meter Hand-held Analyzer Type 2250. The background noise was in accordance with ISO 8253-1 (2010) for all frequencies (in the range 31.5–8,000 Hz) with the highest L_max_ at 55 dB (31.5 Hz). The frequencies selected for audiometry were the following: 250, 500, 1,000, 2,000, 3,000, 4,000, 6,000 and 8,000 Hz. The equipment was calibrated prior to audiometry (ISO 8253-1 2010).

An automated procedure was used, but if there was uncertainty regarding measured hearing thresholds, ongoing tinnitus or former recognized hearing loss, manual audiometry was performed. Individual noise exposure within the last 16 h prior to audiometry was evaluated by a checklist that contained the following choice of statements regarding recent noise exposure: “No loud noise exposure,” “Loud area noise exposure,” “Loud workshop noise exposure” and “Other loud noise exposure.” Navy personnel who reported being highly exposed to noise the previous 16 h (who had stayed in loud area noise; e.g. engine room or workshop) and who had a hearing threshold ≥25 dB in either ear at 3,000, 4,000 or 6,000 Hz were excluded. In order to be included in the study, a new audiometry had to be conducted when they had not been exposed to loud noise the previous 16 h. Audiometry was not performed in cases of present acute airway infections with additional sinus, nose or ear affection, and testing was postponed until the participant became asymptomatic. In cases of inadequate completion of the test protocol, the results were excluded. All results were evaluated by the researcher in cooperation with an otolaryngologist, and referral to supplementary tests was made when indicated.

**Fig. 1:**
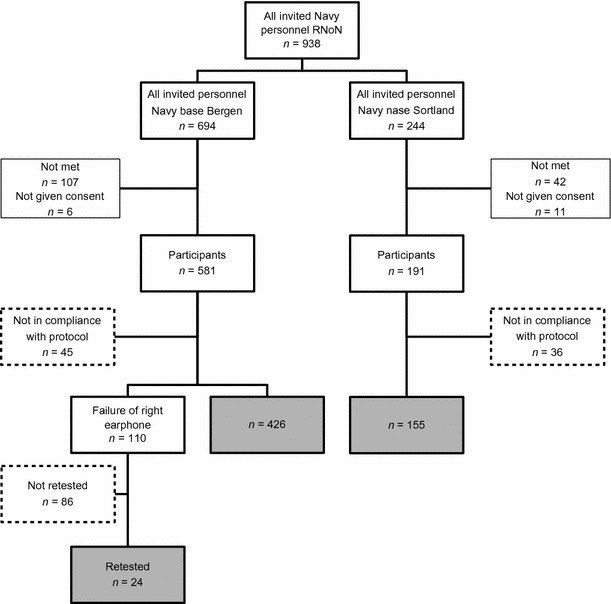
Flowchart describing a study among Navy personnel in Norway, 2012–2013. *Gray boxes* indicate participants included in the analysis (*n* = 605). *Dotted lines* indicate participants excluded from the analysis (*n* = 167)

A total of 772 gave their consent to join the study. In spite of a test regime controlling the audiometer and earphones, an unstable wire connection on the right earphone (Amplivox Audiocups) was discovered after some weeks of testing, affecting 110 measurements conducted in Bergen. In 24 of these cases, an additional audiometry was performed by qualified personnel assigned to the project. We were not able to retrieve the remaining 86 participants for an additional audiometry. Due to this technical failure as well as to insufficient compliance with the test protocol, results from a total of 167 participants had to be excluded, leaving 605 participants included in the study (Fig. 1).

### Analysis

Results are presented as descriptive statistics, using percent, mean, standard deviation (SD) and Pearson correlation coefficient (R).

Participants were categorized into job categories according to working position on board. Hearing loss was defined as hearing threshold levels ≥25 dB in either ear at 3,000, 4,000 or 6,000 Hz. Continuous variables of potential determinants of hearing loss were categorized in quartiles. Log binomial regression provided relative risks (RR) with 95 % confidence intervals (CI) of hearing loss among the different determinants. Only determinants with significant impact on hearing were presented.

In order to adjust for the influence of age and gender on hearing, a new variable was constructed. Based on ISO 7029 (2000), we calculated the age and gender-specific hearing threshold and compared this expected value with the respective participants’ measured hearing thresholds. In this calculation, the 50 percentile hearing threshold provided in the ISO standard was chosen. Deviation from the expected hearing threshold value was calculated as:

$$\Delta \,{\text{Hearing}}\,{\text{threshold}} = {\text{measured}}\,{\text{hearing}}\,{\text{threshold}}\,-\,{\text{expected}}\,{\text{hearing}}\,{\text{threshold}}\,{\text{according}}\,{\text{to}}\,{\text{ISO}}\,7029$$

A Δ Hearing threshold <0 indicated a better hearing than according to ISO 7029, Δ Hearing threshold = 0 indicated hearing equal to ISO 7029, while Δ Hearing threshold >0 indicated poorer hearing than according to ISO 7029. This calculation was made for each frequency for both ears, and for each participant, the poorest Δ Hearing threshold of the two ears for each frequency was chosen.

The association between the work exposure variables (years of work in the Navy, years on vessel(s) in the Navy and years of sailing in the Navy) and Δ Hearing threshold for each frequency was analyzed by linear regression providing β and 95 % CI. The results were adjusted for otitis as an adult, which was the only variable apart from age with significant negative impact on hearing in our data. This analysis was only completed for the 522 participants tested with the Interacoustics AD226 audiometer, thus excluding those who were tested with the audiometer with +10 dB as the minimum test level. In a separate linear regression analysis, we excluded participants with prior otitis as an adult instead of adjusting for this. This analysis was completed for 453 participants tested with the Interacoustics AD226 audiometer.

Statistical significance was set at *p* < 0.05. The data were analyzed using IBM SPSS Statistics, version 21.

### Research ethics

The study was performed in accordance with the 1964 Declaration of Helsinki and its later amendments. The study protocol was approved by the Regional Committees for Medical and Health Research Ethics, REC South East. The participants were informed about the objectives and conditions of the study and gave their informed consent. The participants received no payment for participating in the study, and they could withdraw from the study at any point. Individual data from the study could not be used as a basis for medical selection of candidates. The Royal Norwegian Navy permitted all available results on group level to be published.

## Results

The study population consisted of 605 participants, of which 569 were male and 36 female. The mean age of the participants was 30 years, with a range from 19 to 62 years. A total of 190 participants (31.4 %) had hearing loss defined as hearing threshold levels ≥25 dB in either ear at 3,000, 4,000 or 6,000 Hz.

The prevalence of hearing loss was significantly higher among navigators (37.0 %) and engine room personnel (38.0 %) than electricians (23.6 %) (Table 2).

**Table 2:** Prevalence and relative risk (RR) of hearing loss by job category in a study of 605 Navy personnel in Norway, 2012–2013

Job category	Total number^a^	Mean age (min–max)	Hearing loss^b^	RR	95 % CI
	*n*		*n* (%)		
Electrician	106	28 (20–48)	25 (23.6)	1 (ref)	
Work on deck	88	26 (19–46)	22 (25.0)	1.06	0.64–1.75
Work in ships office	36	29 (20–47)	10 (27.8)	1.18	0.63–2.21
Cook	25	26 (19–53)	8 (32.0)	1.36	0.70–2.64
Work in operation room	118	29 (19–50)	40 (33.9)	1.44	0.94–2.20
Navigator	119	34 (23–62)	44 (37.0)	1.57*	1.04–2.38
Engine room personnel	108	31 (19–54)	41 (38.0)	1.61*	1.06–2.45

Log binomial analysis

* Statistical significance

^a^Job category was missing for five participants

^b^Hearing loss defined as ≥25 dB in either ear at 3,000, 4,000 or 6,000 Hz

The log binomial regression (*n* = 605) showed that hearing loss was significantly associated with age, the work exposure variables: years of work in the Navy, years on vessel(s) in the Navy and years of sailing in the Navy, otitis as an adult, attending concerts/disco and use of Mp3 player (Table 3).

**Table 3:** Significant determinants with effect on hearing, measured in 605 Navy personnel in Norway, 2012–2013

Determinant		Hearing loss	Normal hearing	RR	95 % CI
		*n* (%)	*n* (%)		
Age^b^	<24 years (ref)	42 (23.0)	141 (77.0)	1	
	24–27 years	29 (25.0)	87 (75.0)	1.09	0.72–1.65
	28–33 years	39 (26.5)	108 (73.5)	1.16	0.79–1.69
	>33 years	80 (50.3)	79 (49.7)	2.19*	1.61–2.98
Years of work in the Navy^b^	0–2 years (ref)	30 (21.9)	107 (78.1)	1	
	2.1–5 years	39 (26.2)	110 (73.8)	1.20	0.79–1.81
	5.1–11 years	42 (26.9)	114 (73.1)	1.23	0.82–1.85
	>11 years	78 (48.4)	83 (51.6)	2.21*	1.55–3.15
Years on vessel(s) in the Navy^b^	0–2 years (ref)	49 (24.1)	154 (75.9)	1	
	2.1–4 years	35 (29.7)	83 (70.3)	1.23	0.85–1.78
	4.1–9 years	32 (22.2)	112 (77.8)	0.92	0.62–1.36
	>9 years	73 (52.9)	65 (47.1)	2.19*	1.64–2.93
Years of sailing in the Navy^b^	<1 year (ref)	73 (26.4)	203 (73.6)	1	
	1.1–3 years	28 (21.7)	101 (78.3)	0.82	0.56–1.20
	>3 years	85 (46.4)	98 (53.6)	1.76*	1.37–2.26
Otitis as an adult	No (ref)	139 (29.1)	338 (70.9)	1	
	Yes	39 (50.6)	38 (49.4)	1.74*	1.34–2.26
	I don’t know	12 (24.0)	38 (76.0)	0.82	0.49–1.38
Concerts/disco	Seldom/never (ref)	57 (45.6)	68 (54.4)	1	
	Sometimes/year	76 (29.8)	179 (70.2)	0.65*	0.50–0.86
	≥Sometimes/month	57 (25.6)	166 (74.4)	0.56*	0.42–0.75
Mp3 player	Seldom/never (ref)	95 (38.3)	153 (61.7)	1	
	1–2 h/week	49 (26.3)	137 (73.7)	0.69*	0.52–0.92
	≥3 h/week	46 (27.1)	124 (72.9)	0.71*	0.53–0.95

Log binomial analysis

* Statistical significance

^a^Hearing loss: ≥25 dB in either ear at 3,000, 4,000 or 6,000 Hz

^b^Continuous variables are divided in quartiles

The prevalence of hearing loss was 50.3 % among Navy personnel aged above 33 years, and 23.0 % among those aged below 24 years (Table 3). Navy personnel who had sailed for more than three years in the Navy had a 46.4 % prevalence of hearing loss, while the prevalence was 26.4 % among the Navy personnel who had sailed in the Navy for less than one year (Table 3). However, the work exposure variables were all significantly inter-correlated (Pearson correlation) with age: years of work in the Navy (R = 0.88, *p* < 0.001), years on vessel(s) in the Navy (R = 0.85, *p* < 0.001) and years of sailing in the Navy (R = 0.80, *p* < 0.001). Among the 77 participants who had experienced otitis as an adult, 50.6 % had hearing loss (Table 3). Two determinants were associated with a reduced risk of hearing loss: attending concerts/disco and using Mp3 player (Table 3). No association was observed between hearing loss and the following variables from the questionnaire: impulse noise, use of hearing protection, work with organic solvents, diving, heart disease, hypertension, diabetes, otitis as a child, reduced hearing in closest family, episodes of temporary reduced hearing, admittance to hospital due to head injury, ototoxic medication, use of cigarettes, use of moist snuff, hunting and number of gunshots the previous year or playing in a band (data not shown).

Using the age and gender-adjusted variable Δ Hearing threshold and adjusting for otitis as an adult in a linear regression model (*n* = 522), the hearing threshold level increased significantly for all the three work exposure variables at 1,000 and 4,000 Hz (Table 4). The hearing threshold level at 3,000 Hz was significantly increased only for the work exposure variable years of sailing in the Navy. Among the three work exposure variables, years of sailing in the Navy was the strongest predictor of impaired hearing. There was no statistically significant association between the work exposure variables and hearing threshold levels at 6,000 Hz (a frequency often associated with NIHL), nor at the frequencies 250, 500, 2,000 or 8,000 Hz. In the separate analysis in which participants with prior otitis as an adult were excluded (*n* = 69), the hearing threshold level was significantly increased for the work exposure variable years of sailing in the Navy at 1,000, 3,000 and 4,000 Hz (data not shown). Years on vessel(s) in the Navy was associated with a significantly poorer hearing threshold at 4,000 Hz. Years of work in the Navy was not associated with impaired hearing thresholds in this analysis.

**Table 4:** Age and gender-adjusted (ISO 7029) Δ Hearing threshold related to years of noise exposure among 522 Navy personnel in Norway, 2012–2013

Work exposure	Audiometry frequency (Hz)							
	1,000		3,000		4,000		6,000	
	β	95 % CI	β	95 % CI	β	95 % CI	β	95 % CI
Years of work in the Navy	0.11*	0.02, 0.21	0.05	−0.05, 0.16	0.15*	0.03, 0.28	−0.04	−0.20, 0.12
Years on vessel(s) in the Navy	0.19*	0.07, 0.31	0.10	−0.04, 0.23	0.24*	0.07, 0.40	−0.07	−0.28, 0.15
Years of sailing in the Navy	0.35*	0.17, 0.54	0.26*	0.05, 0.47	0.48*	0.22, 0.73	−0.12	−0.45, 0.21

Adjusted for otitis as an adult

Linear regression analysis with β in dB/year

* Statistical significance

## Discussion

The prevalence of hearing loss among Navy personnel was 31.4 %. Hearing loss was associated with the work exposure variables: years of work in the Navy, years on vessel(s) in the Navy and years of sailing in the Navy, as well as age and otitis as an adult. When adjusting for age, gender and otitis as an adult, higher hearing thresholds at 1,000 and 4,000 Hz were found when assessing the work exposure variables. Of the three work exposure variables, years of sailing in the Navy was the strongest predictor of hearing loss in our study and suggests that work on board RNoN vessels is detrimental to hearing.

Similar results were also found when excluding participants with prior otitis as an adult. However, for years of employment and years on vessel(s) in the Navy, this association was weaker and might be explained by the smaller sample size when excluding those with prior otitis as an adult. Using Mp3 player and attending concerts/disco seemed to have a positive impact on hearing. This finding might be related to the assumption that those who listen to loud music may tolerate the noise exposure, hence not developing hearing loss. Another explanation can be that those who already have developed hearing loss give up attending concerts/disco and listening to Mp3 player in order to avoid further deterioration of hearing. However, usage of Mp3 player and attending concerts was inversely associated with age and years of employment and the observed association may therefore have been confounded. The prevalence of hearing loss was significantly higher among navigators and engine room personnel than among electricians, suggesting that the noise exposure varies with job category.

Hearing loss can be classified in numerous ways, rendering comparison of hearing loss between different studies a challenge (Rabinowitz et al. 2012). Frequencies most important for speech discrimination can be emphasized (e.g., the U.S. Navy), while our definition is based on frequencies associated with NIHL. The U.S. Navy uses “significant threshold shift” (STS), which is defined as a change in hearing threshold relative to the initial reference audiogram of 10 dB or more averaged over 2,000, 3,000, and 4,000 Hz, in either ear (DoDI 6055.12 2013).

As an example, a U.S. study has stated that the STS prevalence would be higher if using the criteria set by the Occupational Safety and Health Administration (OSHA) rather than using those set by the U.S. Navy (Wolgemuth et al. 1995).

The prevalence of hearing loss in the present study was 31.4 %. In a previous study, self-reported hearing loss was prevalent among 24 % of RNoN personnel (Moen et al. 2008). In contrast only 3 % of the population in a national population health survey reported hearing loss (Norway 2003) and the prevalence of disabling hearing loss among inhabitants in a Norwegian county was 10.3 % (Engdahl and Tambs 2010). In studies based on data from the U.S. Navy Hearing Conservation Program, the rate of total STS varied between 18.1 % (Bohnker et al. 2002b) and 29 % (Wolgemuth et al. 1995). The higher prevalence of hearing loss in our study suggests that the hearing loss can be attributed to work on board RNoN vessels.

We found an association between reduced hearing and work on board navy vessels. In a study comparing hearing thresholds in U.S. Navy and Marine Corps with OSHA age-corrected values of hearing thresholds (Bohnker et al. 2002a), it was concluded that men in Navy and Marine Corps had higher threshold levels than according to OSHA. Another study reported that working on board surface warships was more damaging to hearing than work at shore duty stations (Trost and Shaw 2007), and an increased risk of hearing impairment was indicated in a study among flight deck personnel and engine room workers on an aircraft carrier compared with administrative personnel (Rovig et al. 2004). The relationship between noise exposure on board Navy vessels and reduced hearing seen in prior studies is in line with our findings.

In the present study, we found an association between noise exposure and higher hearing thresholds at 1,000–4,000 Hz but not at 6,000 Hz. It has been reported that hearing loss appears differently depending on whether the noise exposure is continuous or results from impulse noise, like explosions and firing cannons. Continuous noise exposure tends to result in notching at 4,000 Hz (McBride and Williams 2001). Two studies which described exposure to acoustic trauma among Finnish conscripts and Finnish surviving suicide bomb victims both found the poorest hearing thresholds at 6,000 Hz (Mrena et al. 2004; Ylikoski 1989). However, this is only partly in line with findings from the larger Norwegian study, which observed approximately equal hearing thresholds at 3,000, 4,000, 6,000, and 8,000 Hz among men exposed to impulse noise (Tambs et al. 2006). Thus, based on prior literature, it is difficult to conclude whether the reduced hearing found in our study is caused by the continuous noise exposure on board or by impulse noise.

The highest prevalence of hearing loss was seen among engine room personnel (38.0 %). Comprehensive noise level measurements on board Navy vessels have barely been reported; however, studies from commercial vessels have shown that noise levels in engine rooms range from around 90 to 110 dB(A) (Neitzel et al. 2006; Svendsen and Børresen 1999; Turan et al. 2011). In a study among Danish seafarers and fishermen, the engine room personnel had a 2.39 times greater risk of hearing loss compared with other seafarers (Kaerlev et al. 2008). In U.S Navy studies the prevalence of STS among enginemen varies between 18.0 and 20.2 % (Bohnker et al. 2002b; Wolgemuth et al. 1995). The high prevalence of hearing loss among engine room personnel seen in our study might be due to high noise levels in engine rooms on board RNoN vessels.

The lowest prevalence of hearing loss in our study was seen among electricians (23.6 %). Noise levels in the engine control room (where electricians have their work site) of ferries, cargo ships and westamarans range from around 70 to 90 dB(A) (Svendsen and Børresen 1999). These levels are lower than the levels in the engine rooms (Neitzel et al. 2006; Svendsen and Børresen 1999; Turan et al. 2011), but may still represent a hazard to hearing for sensitive individuals. Previous studies have also shown that electricians have a low prevalence of hearing loss, even lower than in our study. A U.S. Navy study comparing rates of STS found the lowest value among “Electronics technicians” (5.0 %) (Wolgemuth et al. 1995). Another U.S. Navy study which compared rates of STS among Navy and Marine Corps found STS prevalence ranging from 15.8 to 23.8 % among electrician groups (Bohnker et al. 2002b). The somewhat higher prevalence of hearing loss among electricians in our study might be due to higher noise levels in engine control rooms on board RNoN vessels than in the previously studied vessels.

The response rate in this study was high (81.4 %); however, the participation rate was only 63.8 %. This was due to the fact that data were collected in accordance with a stringent protocol. There is no reason to believe that the excluded participants differ from the ones included. We have limited information about the 149 who did not meet for examination and the 17 who did not give consent to participate; hence, we cannot rule out that these non-responders differed from the responders.

Few previous studies on hearing loss among Navy personnel have provided information on confounding factors that might be responsible for hearing loss (Henselman et al. 1995). In our study, a questionnaire regarding occupational and non-occupational noise exposure and other possible determinants of hearing loss was used, which made it possible to adjust for non-occupational determinants in the analysis.

All invited personnel were informed that individual data would not be used to assess medical skillfulness, with criteria for hearing thresholds that must be fulfilled in order to be allowed work on board. Furthermore, there is no reason to believe that recorded hearing thresholds have been biased by participants striving to get a result adequate to be allowed to work on board.

We chose to use ISO 7029 (2000) as a reference to hearing thresholds in the general population. One alternative could be to age adjust in the log binomial analysis, but this would introduce an over-adjustment, as age and years of noise exposure are closely correlated. Hearing loss is present in the youngest age-group (<24 years), suggesting that hearing loss in this population is probably primarily caused by noise exposure and less by aging. The ISO 7029 consists of a screened population free of all symptoms of ear disease, without obstructing wax and without undue history of noise exposure, hence similar to our population with the sole exception of noise exposure (ISO 7029 2000). We chose to calculate the expected hearing thresholds using the 50 percentile, although one could defend choosing 75 or 90 percentiles (acquiring lower hearing thresholds), as our population was screened before enrollment, and one would expect a better hearing than for the population in general. However, choosing these percentiles would make the difference between estimated and measured hearing thresholds even greater, strengthening the results of our study. An alternative to choosing ISO 7029 as a reference was ISO 1999, data base B (ISO 1999 2013), which is based on a Norwegian population and presents hearing threshold levels as a function of age of an unscreened population. However, as personnel are screened when enrolled in the Navy, we chose to compare with the screened population of ISO 7029 instead. As we wished to adjust hearing thresholds at an individual rather than on a group level, we found that ISO 7029 was the preferable reference material.

Although we found coherence between years of sailing in the Navy and impaired hearing, the cross-sectional study design cannot clarify cause and effect.

In the RNoN today, no definite protocol is established on how to follow up personnel with recognized hearing loss. We hope that this study, stating a high prevalence of hearing loss, will contribute to further awareness of the noise problem on board. Noise measurements and subsequent protection against high noise levels should be implemented, and a hearing conservation program should be established in order to improve working conditions on board. As the population is young, the benefit from prevention is great and hearing can still be protected and preserved.
